# RETRACTION: Hypoxia‐Induced GST1 Exerts Protective Effects on Trophoblasts via Inhibiting Reactive Oxygen Species (ROS) Accumulation

**DOI:** 10.1155/ancp/9852510

**Published:** 2026-06-24

**Authors:** Analytical Cellular Pathology

RETRACTION: L. Chen, G. Chen, L. Guo, Y. Wang, and C. Ai, “Hypoxia‑Induced GST1 Exerts Protective Effects on Trophoblasts via Inhibiting Reactive Oxygen Species (ROS) Accumulation,” *Analytical Cellular Pathology*, 2023, 9391252, https://doi.org/10.1155/2023/9391252.

The above article, published online on 25 January 2023 in Wiley Online Library (http://wileyonlinelibrary.com), has been retracted by agreement between the Journal Editor‐in‐Chief, Dimitrios Karamichos; and John Wiley & Sons Ltd. The retraction has been agreed due to concerns raised with the figures, initially raised on PubPeer [1]. Further investigation identified additional inconsistencies with the figures. A full summary of the concerns can be found below:

• The GSTP1 bands in Figure 2a appear identical to the NDRG1/U87MG bands in Figure 2c of [2]
• The GSTP1 bands in Figure 3a appear identical to the Cleaved PARP1 bands shown in Figure 5c of [3]
• The ß‐actin bands in Figure 3a appear identical to the ß‐actin bands shown in Figure 5c of [3]
• In Figure 5c, there is an unexpected, repeated region shared between the 48 h/Normoxia and Hypoxia/GSTP1 panels
• The hypoxia flow cytometry plot in Figure 5d appears highly similar to the 200 nmol/L DOX & 15 umol/CHE flow cytometry plot in Figure 4b of [4]
• The hypoxia + GSTP1 flow cytometry plot in Figure 5d appears similar to the MG63 CSCs shLRPPRC + Mock flow cytometry plot in Figure 5a of [5]

Concerns were also raised regarding the apoptosis measurements in Figure 5d and the cell viability data in Figure 2c.

The authors did not provide a response to the concerns, and after assessment by the Chief Editor the results and conclusions can no longer be considered reliable. Therefore, the article is retracted.

The authors did not respond to the retraction.
